# Treatment of Peritoneal Surface Malignancies by Cytoreductive Surgery (CRS) and Hyperthermic Intraperitoneal Chemotherapy (HIPEC) in Spain: Results of the National Registry of the Spanish Group of Peritoneal Oncologic Surgery (REGECOP)

**DOI:** 10.3390/jcm12113774

**Published:** 2023-05-31

**Authors:** Israel Manzanedo, Fernando Pereira, Pedro Cascales-Campos, Cristobal Muñoz-Casares, Enrique Asensio, Juan Torres-Melero, Arancha Prada-Villaverde, Ibán Caravaca-García, Alberto Gutiérrez-Calvo, Javier Vaqué, Gloria Ortega, Alberto Titos-García, Laura González-Sánchez, Estíbalitz Pérez-Viejo, Ángel Serrano, Beatriz Martínez-Torres, REGECOP Group

**Affiliations:** 1 Peritoneal Carcinomatosis Unit, Department of General and Digestive Surgery, Hospital Universitario de Fuenlabrada, 28942 Madrid, Spain; fernando.pereira@salud.madrid.org (F.P.); estibalitz.perez@salud.madrid.org (E.P.-V.); aserranom@salud.madrid.org (Á.S.); bmartinezt@salud.madrid.org (B.M.-T.); 2 Department of Surgery, Rey Juan Carlos University (URJC), 28933 Madrid, Spain; 3 Spanish Group of Peritoneal Oncologic Surgery (GECOP), 28001 Madrid, Spain; cascalescirugia@gmail.com (P.C.-C.); fcocris@gmail.com (C.M.-C.); easensiodi@saludcastillayleon.es (E.A.); juantorresmelero@gmail.com (J.T.-M.); aranchaprada@hotmail.com (A.P.-V.); ivan_med06@hotmail.com (I.C.-G.); alberto.gutierrez@salud.madrid.org (A.G.-C.); fjvaque@yahoo.es (J.V.); gortega@mdanderson.es (G.O.); albertotitosg@hotmail.com (A.T.-G.); lgsanchez08@gmail.com (L.G.-S.); 4 Peritoneal Oncologic Surgery Unit, Department of Surgery, Hospital Virgen de la Arrixaca, IMIB-ARRIXACA, 30120 Murcia, Spain; 5 Department of Surgery, Hospital Virgen del Rocío, 41013 Sevilla, Spain; 6 Advanced Oncologic Surgery Unit, Department of General and Digestive Surgery, Hospital Río Hortega, 47012 Valladolid, Spain; 7 Department of General and Digestive Surgery, Hospital Universitario de Torrecárdenas, 04009 Almería, Spain; 8 Department of General and Digestive Surgery, Hospital Infanta Cristina, 06080 Badajoz, Spain; 9 Department of General and Digestive Surgery, Hospital General Universitario de Elche, 03203 Alicante, Spain; 10 Department of General and Digestive Surgery, Hospital Príncipe de Asturias de Alcalá de Henares, 28805 Madrid, Spain; 11 Department of General and Digestive Surgery, Hospital de La Fe, 46026 Valencia, Spain; 12 Department of General and Digestive Surgery, Hospital MD Anderson Cancer Center, 28033 Madrid, Spain; 13 Department of General and Digestive Surgery, Hospital Regional Universitario de Málaga, 29010 Málaga, Spain; 14 Department of General and Digestive Surgery, Hospital Insular, 35016 Las Palmas de Gran Canaria, Spain

**Keywords:** peritoneal carcinomatosis, HIPEC, cytoreductive surgery, Peritoneal Surface Malignancies

## Abstract

Introduction: Treatment of Peritoneal Surface Malignancies (PSM) with cytoreductive surgery (CRS) and hyperthermic intraperitoneal chemotherapy (HIPEC) has achieved results never seen before in these patients, which classically have a poor prognosis. The possibility of conducting clinical trials in these diseases is complicated, since some of them are rare, so the analysis of large databases provides very valuable scientific information. The aim of this study is to analyze the global results of the National Registry of the Spanish Group of Peritoneal Oncologic Surgery (REGECOP), whose objective is to register all patients scheduled for HIPEC nationwide. Methods: This is a retrospective analysis of the data recorded in the REGECOP from 36 Spanish hospitals from 2001 to 2021. There were 4159 surgical interventions in 3980 patients. Results: 66% are women and 34% are men with a median age of 59 years (range 17–86). 41.5% of the patients were treated for Peritoneal Metastases (PM) of colorectal cancer (CRC); 32.4% were women with ovarian cancer (OC) with PM; 12.8% were treated for pseudomyxoma peritonei (PMP); 6.2% had PM from gastric cancer (GC); 4.9% had PM of non-conventional origin; and, finally, 2.1% of cases were patients diagnosed with peritoneal mesothelioma. The median Peritoneal Cancer Index (PCI) was 9 (0–39), and complete cytoreduction was achieved in 81.7% of the procedures. Severe morbidity (Dindo–Clavien grade III–IV) was observed in 17.7% of surgeries, with 2.1% mortality. Median hospital stay was 11 days (0–259). Median overall survival (OS) was 41 months for CRC patients, 55 months for women with OC, was not reached in PMP patients, was 14 months for GC patients, and 66 months in mesothelioma patients. Conclusions: large databases provide extremely useful data. CRS with HIPEC in referral centers is a safe treatment with encouraging oncologic results in PSM.

## 1. Introduction

Peritoneal Surface Malignancies (PSM) are a heterogeneous group of primary tumors like primary peritoneal carcinoma or peritoneal mesothelioma, and peritoneal metastases (PM) from other abdominopelvic tumors by cell dissemination, such as from colon, stomach, or ovarian cancer, or pseudomyxoma peritonei secondary to appendiceal mucinous neoplasia [1,2].

Classically, PSM were considered as terminal diseases and therefore were paired with supportive care and palliative treatments. Since the end of the twentieth century, and especially since the beginning of the twenty-first century, progress has been made in the knowledge of the peritoneum and its diseases, and today PSM are considered as local dissemination that can be treated, in selected cases, with radical intent [3].

The combination of modern systemic chemotherapy with cytoreductive surgery (CRS) and hyperthermic intraperitoneal chemotherapy (HIPEC) is achieving very encouraging results and is already considered the standard treatment in some indications, such as peritoneal pseudomyxoma (PMP) or peritoneal mesothelioma [4,5]; in other indications, such as PM from colorectal, ovarian, or gastric cancers, there is more discussion regarding CRS with HIPEC, but the published studies show promising results [6,7,8,9,10].

The scientific evidence on this treatment is growing. However, there are few multicenter randomized clinical trials, because carrying them out is very difficult, since some of these diseases are very rare and make the recruitment of patients very difficult. For this reason, national prospective registries of large patient series are of great importance in clarifying the potential benefit of these procedures and their real risks.

The Spanish Group of Peritoneal Oncologic Surgery (GECOP) was born in 2007 and gathers all the centers where CRS and HIPEC procedures are performed in Spain. It is currently made up by 39 centers. One of the objectives of the GECOP since its foundation was the creation of a prospective registry of patients treated through CRS and HIPEC. However, it was not until 2020 that this goal became a total reality and the National Registry of the Spanish Group of Peritoneal Oncologic Surgery (REGECOP) began to be fully operational. The objective of this article is to analyze the overall results of the REGECOP.

## 2. Patients and Methods

This is a retrospective, multicenter study from a prospective national database of patients with PSM of different origins scheduled for treatment with CRS and HIPEC from 2001 to 2021. Both patients with PSM and patients at high risk of developing PM and who had undergone second-look surgery with prophylactic HIPEC were included. An intention-to-treat analysis was carried out. The study was approved by the ethics committee in each participating center.

All participating centers are members of the GECOP and are specialized in the treatment of peritoneal oncological disease. Depending on the number of procedures performed, the centers were divided into 2 groups: high-volume centers if they have performed more than 100 procedures or low-volume centers if they have performed less than 100. The high-volume centers are described in Table 1. A total of 36 centers participated in the study.

The extension of peritoneal disease is established according to the Peritoneal Cancer Index (PCI) [11], and the radicality of CRS is assessed by the Completeness of Cytoreduction Score (CCS) [12]. CRS is considered a high-complexity surgery when more than 4 visceral resections or peritonectomies have been performed. Data about HIPEC itself is also collected, such as type of perfusion technique (open close, or close with CO_2_ recirculation), drug used, or administration time.

Postoperative morbidity is classified according to Dindo–Clavien classification [13], and complications grade III or IV are considered as severe morbidity. Hospital stay is defined as the days between date of surgery and date of discharge.

During surveillance, recurrences and deaths are registered. Reverse Kaplan–Meier was used to calculate median follow-up. Disease-free survival (DFS) is defined as the time between the date of surgery and the date of first relapse or death. Overall survival (OS) is defined as the time from the date of surgery to death.

The statistical analysis was performed using IBM SPSS software, version 22.0. Outcome analysis was performed using the Chi square test, *t* test, Mann–Whitney *U* test, and contingency tables. Kaplan–Meier curves were used for survival analysis, the log rank test to identify difference between curves, and Cox multiple regression analysis to investigate possible prognostic factors; *p* ≤ 0.05 is considered significant.

## 3. Results

The 36 centers have registered 4159 procedures in 3980 patients. Repeated CRS with HIPEC was performed in 155 patients: 136 were operated on twice, 15 received CRS and HIPEC three times, 3 patients were operated on four times, and 1 patient five times. Major preoperative, surgical, and postoperative characteristics of the 4159 procedures are summarized in Table 2. A comparison was made between high- and low-volume centers, which is shown in Table 3.

### 3.1. Colorectal Cancer

**Figure 1:**
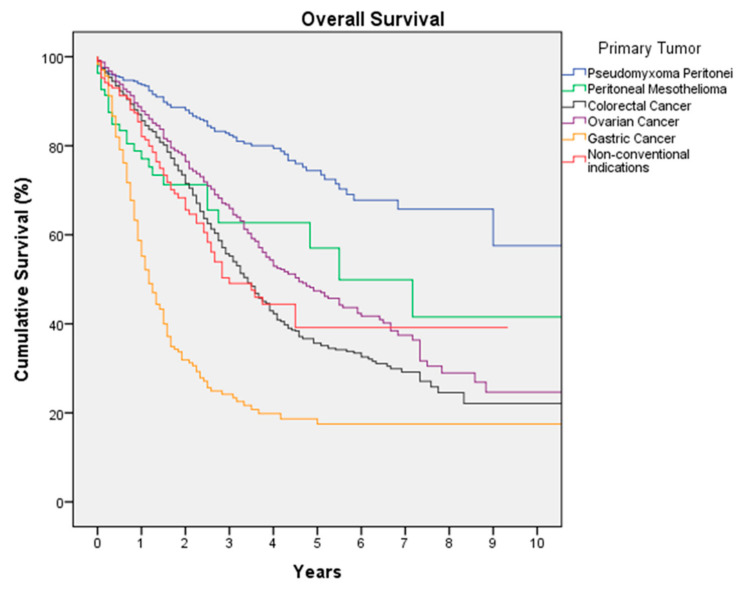
Overall survival of patients treated with cytoreductive surgery and HIPEC according to tumoral origin.

**Figure 2:**
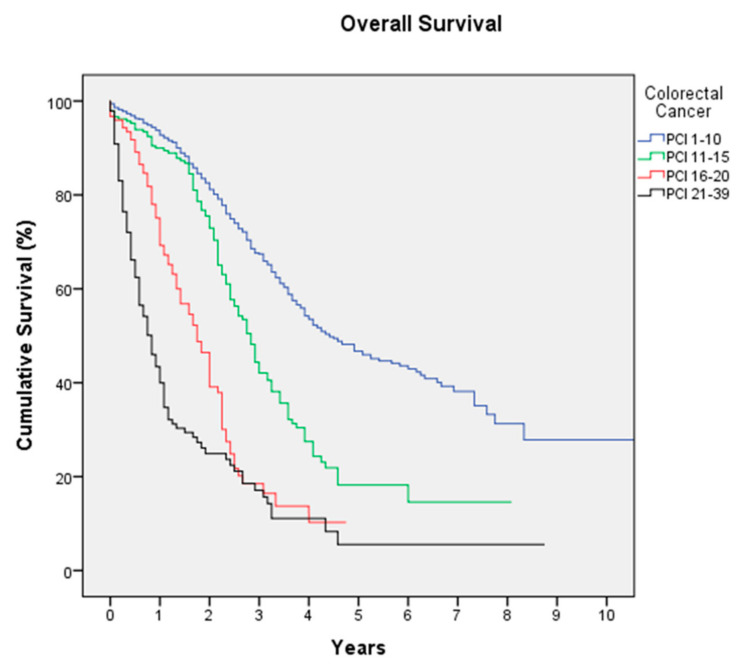
Overall Survival in colorectal cancer according to Peritoneal Cancer Index (PCI).

Colorectal Cancer is the most frequent indication in this registry, with 1716 procedures in 1647 patients. Median age of the patients is 60 years (18–86), with 46.1% women and 53.9% men. Peritoneal relapses (metachronous PM) of a previously operated colorectal cancer constitute 50.8% of the procedures. Preoperative systemic chemotherapy (SCT) was administered in 58.1% of patients. The median surgical PCI is 6 (0–39); 65% of patients have a PCI lower than 10 and 10.8% have a PCI of 20 or higher. Complete cytoreduction (CCS-0) was achieved in 85.5% of surgeries, with a rate of high-complexity surgeries of 32.9%. The most-used drug in HIPEC was mitomycin C (MMC) in 55.2% of cases, followed by oxaliplatin with 41.4%; since June 2018, the use of oxaliplatin dropped significantly, going from being the majority with 56.1% of HIPEC to being used only in 17.2% of cases since then (*p* < 0.05). Severe morbidity occurred in 18.8% of cases, surgical reintervention was necessary in 14.5%, and mortality was 2.5%. The use of oxaliplatin was associated with significant increased severe morbidity (26.2%) compared to the use of MMC (14.4%), accompanied by a higher risk of mortality (4% versus 1.3%, *p* < 0.05). Median hospital stay is 12 days (1–195). With a median follow-up of 37 months, median DFS is 13 months (3-year DFS of 23.9% and 5-year DFS of 17.5%) and median OS is 41 months (3-year OS of 55.7% and 5-year OS of 35.6%) (Figure 1). According to the PCI, median OS is 53 months for a PCI of 0-10, 34 months for a PCI of 11–15, 21 months for a PCI of 16–20, and 10 months for a PCI higher than 20 (*p* = 0.0001) (Figure 2).

### 3.2. Ovarian Cancer

One thousand three hundred twenty-four CRS and HIPEC procedures were performed in 1285 patients with ovarian cancer and PM. The median age is 59 years (20–85). The majority of procedures are primary cytoreductions (68.1%), with 31.9% being secondary cytoreductions (relapses surgery). Upfront surgery (CRS without neoadjuvant SCT) was carried out in 10% of primary cytoreductions; 90% of primary cytoreductions were interval surgeries (CRS after neoadjuvant SCT). The median PCI is 11 (0–39); 23.7% of cases have a PCI higher than 20. Complete cytoreduction (CCS-0) was achieved in 82.4% of cases and CCS-1 in 9.4%, with a rate of high-complexity surgeries of 54.6%. The most-used HIPEC drug was paclitaxel (52.9%), followed by cisplatin (38.9%). Severe complications were observed in 15.6% of patients, and 9.1% of patients were reoperated on in the postoperative period; postoperative mortality was 1.3%. The median hospital stay is 10 days (1–160). The median follow-up is 34 months. The median DFS is 16 months (3-year DFS of 31.1% and 5-year DFS of 24.1%). The median OS is 55 months (3-year OS of 66.7% and 5-year OS of 47.4%) (Figure 1). Median OS in patients with a PCI of 0-20 was 66 months, and it was 29 months in those with a PCI higher than 20 (*p* = 0.0001). Complete cytoreduction (CCS-0 and CCS-1) is a good prognostic factor of OS, with a median OS of 62 months versus 12 months with incomplete CRS (CCS-2 or CCS-3) (*p* = 0.0001).

### 3.3. Pseudomyxoma Peritonei (PMP)

Five hundred and fifty CRS and HIPEC procedures were carried out in 508 patients diagnosed with PMP. Median age of the patients is 60 years (18–85), with 58.9% women. The median PCI is 14 (0–39), significantly higher than the other indications (median PCI of 8, *p* < 0.05); furthermore, 39% of patients have extensive disease, with a PCI greater than 20. Complete cytoreduction (CCS-0) was achieved in 76.1% of surgeries, and nearly complete cytoreduction (CCS-1) in 13.1%. High-complexity surgery was necessary in 47.2% of CRSs. MMC is the HIPEC drug most commonly used (62.6%), followed by oxaliplatin (30.4%). Severe morbidity was observed in 19.7% of cases, with a 13.3% reoperation rate in the postoperative period; postoperative mortality is 3.6%. The median hospital stay is 12 days (0–259). The median surveillance is 33 months. The median DFS is 68 months (5-year DFS of 51.9%), and the median OS was not reached (5-year OS of 74.4%) (Figure 1).

### 3.4. Gastric Cancer

**Figure 3:**
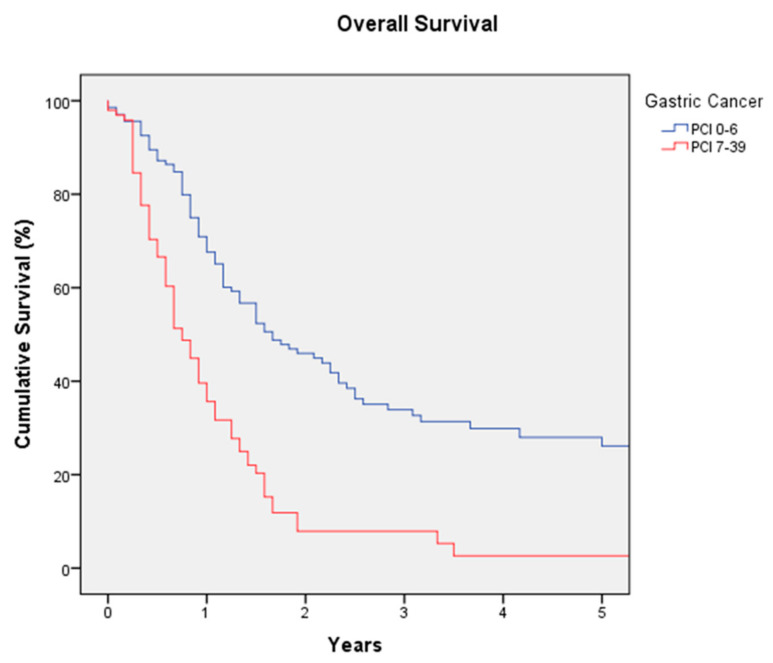
Overall survival in gastric cancer according to Peritoneal Cancer Index (PCI).

Two hundred and fifty procedures of CRS and HIPEC were performed in 246 patients with gastric cancer. The median age of the patients is 56 years (21–84), with 46.2% women and 53.8% men. Neoadjuvant SCT was administered in 89% of patients. The median PCI is 6 (0–39), and 76% of patients have a PCI lower than 12. Complete cytoreduction (CCS-0) was achieved in 77.3% of surgeries, with a high-complexity surgery rate of 41.2%. The HIPEC drug most commonly used was cisplatin alone or in combination with other drugs (61.6%). Severe morbidity was observed in 19% of cases, with 1.7% mortality; the surgical reintervention rate is 10.1%. The median hospital stay is 12 days (1–228). The median follow-up is 40 months. The median DFS is 7 months (3-year DFS of 15.1% and 5-year DFS of 12.3%). The median OS is 14 months (3-year OS of 24.2% and 5-year OS of 18.7%) (Figure 1); the median OS reaches 20 months for a PCI of 0-6 with a 5-year OS of 28%, while for a PCI higher than 6, the median OS falls to 9 months with a 5-year OS of 2.6% (*p* = 0.0001) (Figure 3).

### 3.5. Peritoneal Mesothelioma

One hundred and seven procedures were performed in 90 patients diagnosed with peritoneal mesothelioma. Patients’ median age is 54 years (18–83), with 59.8% women. The median PCI is significantly higher than other indications (20 versus 8, *p* < 0.05), with a PCI ≥ 20 in 51.4% of cases. Complete or nearly complete cytoreduction (CCS-0 or CCS-1) was achieved in 74.2% of CRSs. High-complexity surgery was necessary in 59.6% of CRSs. Cisplatin (alone or in combination) is the HIPEC drug most used (85.3%). Severe complications occurred in 16.8% of cases, with an 11.1% surgical reintervention rate; postoperative mortality is 2.8%. The median hospital stay is 11.5 days (1–142). The median follow-up is 23 months. The median DFS is 11 months (3-year DFS of 27.2%), and the median OS is 66 months (3-year OS of 62.7%) (Figure 1).

### 3.6. Non-Conventional Indications

One hundred and ninety-nine CRS and HIPEC procedures were carried out in 194 patients. The origins were very diverse, with the most frequent being endometrial and small bowel cancer, non-mucinous appendix neoplasms, or sarcomas. The median age is 56 years (18–81), with 68.8% women. The median PCI is 9 (0–39). Complete cytoreduction (CCS-0) was reached in 81.6% of CRSs, with a high-complexity surgery rate of 42.3%. Severe morbidity was registered in 14.9% of cases, and the mortality rate was 0.5%. Median hospital stay is 10 days (1–67). The OS results are shown in Figure 1: the median DFS is 12 months (5-year DFS of 20.9%), and the median OS is 36 months (5-year OS of 39.2%).

## 4. Discussion

The management of PSM has changed in recent years, from being terminal diseases with a poor prognosis to diseases with curative treatment possibilities in selected cases. CRS associated with HIPEC have achieved encouraging results in different tumoral origins. However, the medical community remains skeptical and critical of this treatment due to the few published randomized clinical trials to date, although several clinical trials have been published and multiple studies are ongoing [6,7,8,9,14,15,16,17,18]. Currently CRS with HIPEC is considered the standard treatment only for PMP and peritoneal mesothelioma, curiously two pathologies where there are no published clinical trials because they are rare diseases [19,20]. While scientific evidence continues to grow, national prospective registries with large numbers of patients can contribute to clarify the benefit of these procedures and their efficacy by analyzing morbidity and mortality.

This study shows the results of a large database of Spanish centers. It is one of the studies with the largest number of patients that have been published to date. The main indications are colorectal cancer and ovarian cancer (more than 70%), because these are the most prevalent diseases. Despite the fact that surgical technique is demanding, with a high-complexity surgery rate greater than 40% to achieve a high percentage of complete cytoreductions (81.7%), serious morbidity is quite acceptable, with 17.7%, and 2.1% mortality. Although morbidity is lower in high-volume centers (Table 3), this improvement is at the expense of mild complications, as severe morbidity in low-volume centers is also acceptable. These results are remarkable compared to published studies. In a recent study, Ramos et al. described 20% of severe complications in 1321 consecutive CRS + HIPEC procedures [21]. In 2022, Filis et al. published a meta-analysis in ovarian cancer; they observed 143 adverse events in 308 patients (46.4%) treated with CRS and HIPEC [22]. The PRODIGE 7 trial, published in 2021, registered severe morbidity in 42% of patients in the CRS plus HIPEC group [6].

Colorectal cancer is the most common origin of PM. In the beginning of the 21st century, Verwaal et al. published the first clinical trial of PM of colorectal cancer origin [23]; they randomized 105 patients to receive standard treatment with SCT or aggressive cytoreduction and HIPEC. The median OS in the HIPEC group was significantly higher than the standard group (22.3 months versus 12.6, *p* = 0.032). These results meant a big change, and CRS with HIPEC began to be considered as a treatment option in selected patients. In 2008, the same authors published an update of the trial with more years of follow-up, which confirmed the results obtained in the first study [15]. However, many medical oncologists considered the Verwaal trial quickly outdated since the SCT used in it became obsolete coinciding with the time of the first publication, son, in any case, it would be necessary to make a comparison with the newer drugs (oxaliplatin, irinotecan, cetuximab, and bevacizumab). To answer these questions, the PRODIGE 7 study was carried out; Quenet et al. randomized 265 patients in two groups (CRS alone versus CRS + HIPEC), and the median OS was similar in both groups (41.7 months in the CRS group and 41.2 in the CRS + HIPEC group) [6]. The results of PRODIGE 7 shows that selected patients with PM of colorectal cancer origin must be operated on, because CRS achieves a median OS longer than 3 years, better than what is achieved with SCT, but the addition of oxaliplatin HIPEC is not shown to increase survival; the role of HIPEC is in question, and future studies are necessary to know its value. HIPEC can prevent peritoneal recurrence in locally advanced colorectal cancer, according to a recent trial [24], but its efficacy for the treatment of peritoneal metastases will be evaluated in ongoing studies [7]. The results of the REGECOP demonstrate the efficacy of CRS and HIPEC, with a median OS of 41 months and a 5-year OS of 35.6%; even if patients have a PCI of 10 or lower, the median OS reaches 53 months. Since the results of the PRODIGE 7 study were shown in 2018 in an ASCO meeting, there has been a change in the trend in the REGECOP groups, with using MMC more frequently with the use of oxaliplatin being almost anecdotal at present. On the other hand, our study shows greater severe complications with the use of oxaliplatin. For these reasons, the GECOP group is currently conducting a clinical trial using HIPEC with MMC [7], and currently the HIPEC scheme with oxaliplatin should only be used within clinical trials in view of the results.

Patients with ovarian cancer frequently develop PM, and the use of HIPEC has been controversial. This study shows a median OS of 55 months with a 5-year OS of 47.4%. The most important prognostic factors are the PCI and the quality of cytoreduction according to the CCS; the median OS for a PCI of 0–20 is 66 months (29 months in PCI 21–39), and the median OS is 62 months for CCS-0 or CCS-1 versus 12 months in CCS-2 or CCS-3. The CRS is a standard of care in ovarian cancer, but the use of HIPEC is discussed. Spiliotis et al., in 2015, published a clinical trial for recurrent ovarian cancer; the addition of HIPEC improved OS (3-year OS of 75% versus 18%) in 120 randomized patients [14], but this trial was widely criticized for its methodology. In 2018, Van Driel et al. randomized 245 patients with stage III ovarian cancer, after induction of SCT, to receive interval CRS with or without HIPEC; HIPEC improved median OS (48 versus 34 months) with comparable severe morbidity in both groups [8]. These results are comparable to the trial of Cascales-Campos et al. published in 2022, with an improvement in median OS from 45 months in the non-HIPEC group to 52 months in the HIPEC group without morbidity differences [18]. Despite all the scientific evidence, the gynecologic oncology community remains reluctant to use HIPEC. According to the evidence of these trials and a recent meta-analysis published [22], in primary ovarian cancer the interval CRS with HIPEC is a safe option that improves DFS and OS.

Pseudomyxoma peritonei (PMP) is a rare peritoneal disease originated from a mucinous neoplasm of the appendix. PMP incidence is estimated at around one to three cases per million each year. CRS with HIPEC is the standard treatment with good results. Our study shows the best survival results in PMP despite the fact that the volume of peritoneal disease was high (median PCI of 14), with a DFS of 68 months (5-year DFS of 51.9%) and the median OS not reached (5-year OS of 74.4%). These results are similar to different published studies. In 2021, Kusamura et al. published the results of 1924 patients from the Peritoneal Surface Oncologic Group International (PSOGI) registry; patients treated with CRS and HIPEC had a 5-year OS of 57.8%, better than the patients treated with CRS alone (5-year OS 46.2%) [25]. In 2021, an international consensus was published where bases on the diagnosis and treatment of PMP were founded, establishing CRS and HIPEC as its standard treatment [19].

Gastric cancer with PM has a poor prognosis, with a median OS of 6 months and a 5-year OS of 0% [26]. However, treatment with CRS and HIPEC in selected cases obtains encouraging results. In 2011, a randomized clinical trial was published with 68 patients randomized to CRS and HIPEC or CRS alone; patients treated with HIPEC had significantly better survival (median OS of 11 months versus 6.5 months) [9]. This is the only clinical trial published to date, but there are numerous high-quality studies heading in the same direction. Bonnot et al. published the CYTO-CHIP study in 2019, a propensity score study that compared 180 patients treated with CRS and HIPEC with 97 patients treated with surgery alone; median OS was higher in the HIPEC group (18.8 months versus 12.1), without differences in morbidity or mortality [10]. Furthermore, several meta-analyses showed similar results, with a robust benefit of treatment with CRS and HIPEC [27,28,29,30]. The results of the REGECOP show a median OS of 14 months (5-year OS of 18.7%); if the PCI is lower than 7, the median OS is 20 months (5-year OS 28%). Patient selection is essential to obtain the best results, and experts currently recommend a PCI limit of 12 or even 7 to perform an aggressive treatment with CRS and HIPEC [26,31,32].

Peritoneal mesothelioma is the other established indication for CRS and HIPEC in addition to PMP [20]. In 2013, Baratti et al. published a series of 108 patients with peritoneal mesothelioma treated with CRS and HIPEC with a median OS of 63.2 months [33]. Helm et al. published an important meta-analysis including 1407 patients, and they observed a 5-year OS of 42% [34]. The median OS for peritoneal mesothelioma in our study is 66 months, similar to published studies.

CRS and HIPEC have been used in other non-conventional indications. These have included a miscellany of diverse origins, such as endometrial cancer, small bowel cancer, or sarcomas. This heterogeneity of pathologies makes it impossible to make an analysis of survival, but it is possible to draw conclusions about the safety of the procedure according to morbidity. In the present study, severe morbidity was observed in 4.9% of cases, and the mortality rate was 0.5%. Retrospective studies have been published evaluating the use of CRS and HIPEC as treatment for PM of non-conventional origins. In 2022, Rajha et al. showed the results of 76 patients with PM arising from infrequent tumor entities, with a median OS of 68 months [35]. CRS with HIPEC can be a treatment option for selected patients with PM of different origins.

Despite the obvious limitations of a non-randomized, non-experimental, and solely observational study, this is a study with a large number of patients that provides great information. While waiting for the results of important clinical trials in progress, large national series, such as the present REGECOP study, can be very useful. With the results obtained in this study, we can affirm that CRS with HIPEC is a safe treatment for patients with PSM, which achieves very good outcomes if it is performed in experienced centers. In the coming years, the scientific evidence will increase as different ongoing trials are published [7,17]. Meanwhile, it is very important that these patients are evaluated and treated in expert centers and following the recommendations of different guidelines and consensus [19,20,36,37,38].

## 5. Conclusions

The REGECOP study confirms that treatment with CRS + HIPEC is a safe option for selected patients with PSM. The survival results are remarkable and similar to previously published studies. It is one of the series with the largest number of patients published to date, so our results add highly valid and real information about patients treated in Spain. CRS with HIPEC is the treatment that provides the greatest survival today for patients with PSM. All patients diagnosed with PSM should be referred to experienced centers.

## Figures and Tables

**Table 1:** Centers with more than 100 procedures (high-volume centers).

Hospital	Region	Number of Procedures
H. Universitario de Fuenlabrada	Madrid	735
H. Virgen de la Arrixaca	Murcia	369
H. Virgen del Rocío	Sevilla	271
H. Río Hortega	Valladolid	210
H. Torrecárdenas	Almería	202
H. Infanta Cristina	Badajoz	147
H. General de Elche	Alicante	136
H. Príncipe de Asturias	Madrid	135
H. La Fe	Valencia	129
MD Anderson	Madrid	119
H. Regional de Málaga	Málaga	115
H. Insular	Gran Canaria	102

**Table 2:** Preoperative, surgical, and postoperative characteristics.

Variable	Procedures (*n* = 4159)
Sex (%)	
Female	65.8
Male	34.2
Median age (years (range))	59 (18–86)
Primary tumor (%)	
Colorectal cancer	41.4
Ovarian cancer	31.9
Pseudomyxoma peritonei	13.3
Gastric cancer	6
Peritoneal mesothelioma	2.6
Non-conventional indications	4.8
Neoadjuvant SCT (%)	63.2
Laparoscopic surgery (%)	2.7
Median surgical PCI (range)	9 (0–39)
High-complexity surgery (%)	41.4
CCS (%)	
CCS-0	81.7
CCS-1	7.5
CCS-2	2.2
CCS-3	8.6
HIPEC technique (%)	
Open or coliseum	69.2
Close	3.5
Close with CO_2_ recirculation	27.3
HIPEC drug (%)	
MMC	34.3
Oxaliplatin	24.5
Paclitaxel	18
Cisplatin	10.8
Cisplatin + Doxorubicin	8.4
Cisplatin + MMC	2.5
Others	1.6
Postoperative complications (%)	
No complication	49.9
Minor (I–II)	30.3
Severe complications (III–IV)	17.7
Grade V	2.1
Surgical reintervention (%)	11.9
Median hospital stay (days (range))	11 (0–259)

SCT, systemic chemotherapy; PCI, Peritoneal Cancer Index; CCS, Completeness of Cytoreduction Score; MMC, mitomycin C.

**Table 3:** Comparison of high- and low-volume centers.

Variable	High-Volume Center	Low-Volume Center	*p*
Median surgical PCI (range)	9 (0–39)	8 (0–39)	0.001
CCS (%)			
CCS-0 or CCS-1	89.4	88.9	0.36
CCS-2 or CCS-3	10.6	11.1	
Postoperative complications (%)			
No complication	52.8	44.8	
Minor (I–II)	27.5	35.3	0.0001
Severe complications (III–IV)	17.7	17.5	
Grade V	2	2.4	
Median disease-free survival (months)	16	15	0.49
Median overall survival (months)	47	49	0.48

PCI, Peritoneal Cancer Index; CCS, Completeness of Cytoreduction Score.

## Data Availability

The data used to support the findings of the present study are available from the corresponding author upon request.
